# Occlusal function and masticatory efficiency of sagittal malocclusions: a cross-sectional cohort study of orthognathic therapy

**DOI:** 10.1186/s13005-026-00640-y

**Published:** 2026-07-03

**Authors:** L-M Mai, J Mouchoux, P Meyer-Marcotty, A Quast, P Brockmeyer, H Schliephake, J Schmid, B Wiechens

**Affiliations:** 1 Department of Orthodontics, University Medical Center Göttingen, Göttingen, Germany; 2 Department of Oral and Maxillofacial Surgery, University Medical Center Göttingen, Göttingen, Germany; 3 Department of Orthodontics, University of Münster, Münster, Germany

**Keywords:** Orthognathic surgery, Orthodontics, Dentofacial deformity, Mastication, Dental occlusion

## Abstract

**Background:**

This cross-sectional study aimed to evaluate occlusal characteristics and masticatory performance in patients with skeletal Class II and Class III malocclusions. Both digital occlusal analysis and standardized bolus analysis were employed to assess functional outcomes in non-surgically treated, surgically treated, and control patients.

**Methods:**

A total of 133 orthognathic patients (71 female; median age 29.8 years) and 20 controls (9 female; median age 30.0 years) were included. Skeletal malocclusions were categorized as compensated, decompensated, and a separate surgically treated cohort assessed at 5-year postoperative follow-up. Digital occlusal parameters (total tooth contact (TTC), time of occlusion (TOC), occlusal asymmetry (OAS), anterior and posterior tooth contact (ATC, PTC)) were recorded and masticatory performance was assessed using a standardized two-color bolus analysis.

**Results:**

Non-surgically treated Class II and Class III patients showed significantly reduced TTC, ATC, PTC, and prolonged TOC as well as lower bolus mixing scores compared with controls (*p* < 0.05). Surgically treated patients at 5-year follow-up exhibited occlusal parameters and bolus mixing scores comparable to controls, indicating normalization of occlusal parameters and masticatory efficiency.

**Conclusion:**

Skeletal Class II and Class III malocclusions are associated with impaired occlusal function and reduced masticatory efficiency. Orthognathic therapy effectively restores occlusal parameters as well as functional chewing ability in the long term. Combining digital occlusal analysis with bolus analysis provides a comprehensive and objective evaluation of masticatory rehabilitation in orthognathic patients.

**Trial registration:**

DRKS00025729

## Introduction

The correction of malocclusions represents one of the central components in restoring masticatory function in orthognathic patients [1, 2] with the primary therapeutic aim of orthognathic therapy to improve masticatory performance. This is not only achieved by establishing a precisely adjusted dental occlusion but also an individually aligned skeletal relationship of the jaws [3]. Insufficient preoperative correction of incisal segments can complicate skeletal repositioning, highlighting the need for proper occlusal alignment [4].

For evaluating masticatory and occlusal function, several non-invasive tools are available. This includes not only particle assessment, sieve analyses and dye-release methods but also methods like color-changing chewing gums [5, 6]. Although these traditional techniques provide detailed insights, they are difficult to implement in clinical practice [5–8]. Further, the use of articulating paper is prone to errors due to saliva contamination and false-positive contact marks [9].

With the development of digital measurement systems, it is now possible to assess objective occlusal parameters with higher reproducibility and sensitivity than the traditional techniques [10, 11]. Hereby, the electronic occlusal sensors enable quantification of total contacts and occlusion time, which are one of the key determinants of masticatory performance [2, 10–15].

Meta-analytical evidence shows that masticatory performance improves after orthognathic surgery, with the functional outcomes often remaining below the level observed in patients with physiological occlusion [16]. Different clinical studies have also demonstrated that reduced antagonism as well as increased occlusion times are closely associated with compromised masticatory performance [2, 14].

Furthermore, the relevance of digital analysis of the occlusion is underlined in recent literature. Maurya et al. demonstrated that occlusal force distribution and efficiency significantly improved from pre- to post-treatment in orthodontic patients [17]. Similarly, cross-sectional investigations revealed significant differences in occlusal force distribution between Angle Class I, II, and III malocclusions, which highlights the diagnostic and evaluative value of digital occlusal analysis [18].

As already mentioned, masticatory performance represents a key functional outcome of orthognathic therapy in addition to static occlusal parameters. A recent study by Hamdi et al. has shown, by using a two-colored chewing gum analysis, that chewing efficiency is significantly lower in patients with Angle Class II malocclusion compared to controls, with a significant correlation existing between occlusal contacts and masticatory efficiency [19]. In addition, Reda et al. indicated that functional tooth units are a major determinant of masticatory performance, underlining the functional relevance of occlusal rehabilitation [20].

The relevance of digital occlusal analysis specifically in orthognathic patients was further demonstrated by Wiechens et al., who evaluated patients before and nine months after surgery. Before treatment, patients showed significant deficits in total antagonism, occlusion time, occlusal asymmetry, and contact distribution, while nine months postoperatively, substantial improvements in occlusal function were observed [21, 22].

However, with most existing studies focusing only on short-term outcomes and assessing either occlusal parameters or masticatory performance in isolation, data on occlusal function and masticatory performance several years after orthognathic treatment are limited. Further, long-term facial stability studies showed a tendency toward preoperative morphology [23], underlining the need for extended follow-up to fully capture functional adaptation.

Therefore, the present cross-sectional study aims to assess occlusal characteristics using digital occlusal analysis in combination with standardized two-color bolus analysis to quantify masticatory performance in patients with skeletal Class II and Class III malocclusions. Importantly, this study includes a cohort of treated orthognathic patients examined at a 5-year follow-up, allowing insight into long-term functional outcomes.

## Materials and methods

This study was conducted in accordance with the principles of the Declaration of Helsinki and is listed in the German Clinical Trials Register (number DRKS00025729) with all patients voluntarily participating in the trial after receiving comprehensive information about the study protocol and signing an informed consent form. All participants provided written informed consent prior to participation in the study and clinical examination. The Institutional Ethics Committee of the University Medical Center Göttingen (ethics number 7/1/16) approved this study.

### Patients

**Fig. 1:**
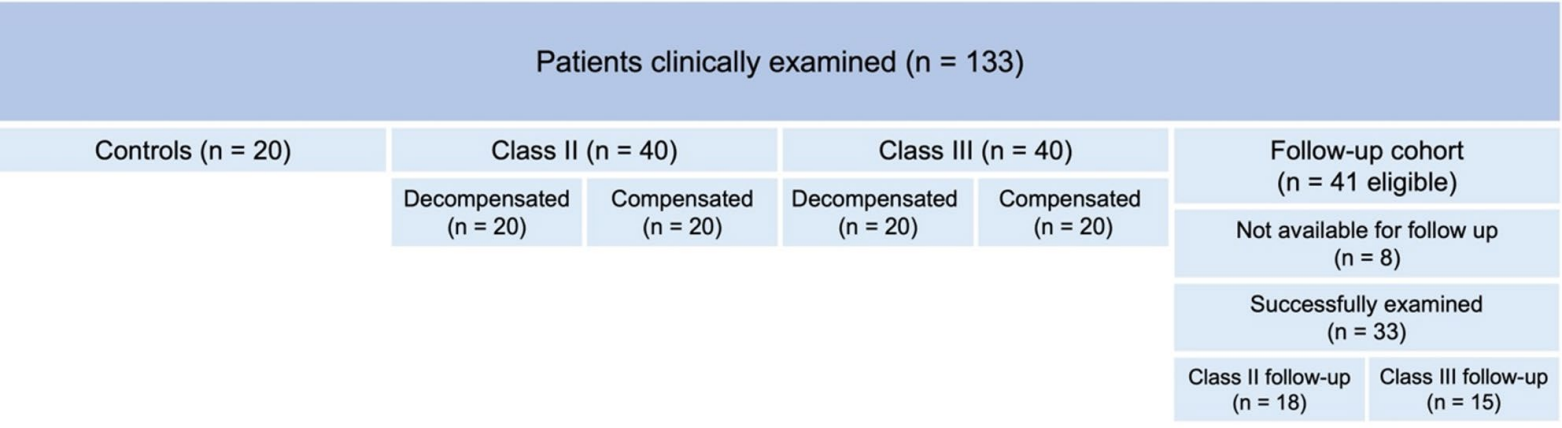
Flowchart of clinically examined patients

One hundred thirty-three orthognathic patients (all ≥ 18 years; 71 women; median age 29.78 years (IQR = 11.12)) participated and were categorized into predefined cohorts based on skeletal classification and treatment status (Fig. 1; Tables 1 and 2). Consecutive patients were prospectively recruited and clinically examined for this study between October 2023 and August 2025.

**Table 1:** Descriptive statistics of the study patients (class II and III malocclusion)

Characteristics of the study patients		Controls I (*N* = 20)				Class II (*N* = 40)								Class III (*N* = 40)							
						Compensated (*N* = 20)				Decompensated (*N* = 20)				Compensated (*N* = 20)				Decompensated (*N* = 20)			
	Unit	Median (IQR)	Min		Max	Median (IQR)	Min		Max	Median (IQR)	Min		Max	Median (IQR)	Min		Max	Median (IQR)	Min		Max
Age	y.m	30.00 (8.00)	23.00		42.00	25.10 (8.03)	18.00		50.00	31.20 (11.70)	18.50		54.10	19.30 (3.40)	18.00		35.50	26.15 (7.25)	19.70		42.00
ANB	°	3.00 (2.25)	1.00		7.30	8.15 (1.35)	4.30		13.90	7.25 (3.78)	2.40		11.50	0.40 (5.93)	-6.40		2.70	1.65 (5.45)	-6.80		5.20
Wits	mm	0.00 (1.55)	-2.00		2.00	5.30 (2.95)	2.05		13.10	4.90 (4.78)	2.02		9.10	-9.70 (10.60)	-16.60		-2.20	-6.00 (5.78)	-17.50		-2.20
ML-NL	°	24.00 (8.00)	12.20		35.00	32.15 (11.60)	11.20		52.10	34.50 (15.13)	12.30		47.00	26.90 (3.80)	9.70		41.80	28.55 (4.38)	14.00		43.80
Overjet	mm	2.30 (1.45)	1.30		4.90	6.40 (4.30)	3.70		15.40	7.70 (3.60)	1.90		11.60	-2.25 (3.38)	-11.30		1.60	-1.85 (2.60)	-13.20		1.90
Overbite	mm	2.20 (1.93)	0.00		5.00	-0.7 (3.78)	-7.60		6.00	0.00 (5.08)	-9.80		6.00	-0.20 (5.20)	-4.50		8.40	-1.25 (2.23)	-4.50		1.90
		female		male		female		male		female		male		female		male		female		male	
Sex	(N)	9		11		13		7		14		6		6		14		10		10	

The median and the interquartile range (IQR) are reported

**Table 2:** Descriptive statistics of the study patients (follow-up)

Characteristics of the study patients		Class II Follow-Up (*N* = 18)				Class III Follow-Up (*N* = 15)			
	Unit	Median (IQR)	Min		Max	Median (IQR)	Min		Max
Age	y.m	34.90 (15.50)	22.20		54.70	29.00 (5.40)	18.10		44.30
ANB	°	8.20 (2.95)	3.80		13.00	1.00 (3.45)	-4.50		2.40
Wits	mm	4.70 (4.30)	2.01		11.00	-5.20 (3.40)	-9.50		-3.60
ML-NL	°	30.75 (11.78)	18.60		41.60	28.90 (10.70)	15.50		39.70
Overjet	mm	8.25 (2.50)	2.10		10.50	-1.00 (2.40)	-3.70		1.60
Overbite	mm	1.25 (2.63)	-3.00		8.30	0.00 (2.35)	-5.80		2.50
		female		male		female		male	
Sex	(N)	12		6		7		8	

The median and the interquartile range (IQR) are reported. The values represent the preoperative measurements obtained prior to orthognathic surgery

The malocclusion cohorts included patients with skeletal Class II or Class III malocclusion and were differentiated according to the stage of treatment. Patients were categorized as dysgnathic compensated (pre-treatment) or orthodontically decompensated Class II or Class III malocclusions (pre-surgical). A further cohort included patients with former skeletal Class II or Class III malocclusion who had undergone orthognathic treatment and were examined at a follow-up of 5 years after completion of surgical therapy treated by the Department of Oral and Maxillofacial Surgery and the Department of Orthodontics at the University Medical Center Göttingen.

The inclusion criteria were skeletal Class II and Class III jaw relationships with indication for orthognathic surgery, defined by the initial Wits appraisal (Class II: Wits > + 2 mm; Class III: Wits < -2 mm). Classification was based on skeletal diagnosis rather than dental occlusal relationships. Exclusion criteria were congenital syndromes, a history of cleft lip and palate, trauma, or previous orthognathic surgery, except in the follow-up cohort. A total of 41 patients were eligible for inclusion in the 5-year follow-up cohort. Of these, 33 participated in the clinical examination, whereas 8 patients could not be recruited for follow-up assessment. At the 5-year follow-up, all surgically treated patients had achieved and maintained Class I occlusion.

The 20 control patients (9 female; median age 30.00 years (IQR = 8.00)) were recruited from a pool of patients with neutral skeletal relation from the Department of Orthodontics. All control patients had a neutral jaw relationship (Wits), Class I occlusion and complete dentition of natural teeth (no implants or prosthetic restorations; if a symmetric class-I-relation was present, premolar agenesis was not considered an exclusion or inclusion criterion). The exclusion criteria for controls were the presence of temporomandibular disorders, midline deviations, crossbites, or non-occlusions. The presence of temporomandibular disorders was assessed clinically using a standardized functional examination including assessment of joint sounds, mandibular movement, pain on palpation, and functional limitations.

### Occlusal registration

**Fig. 2:**
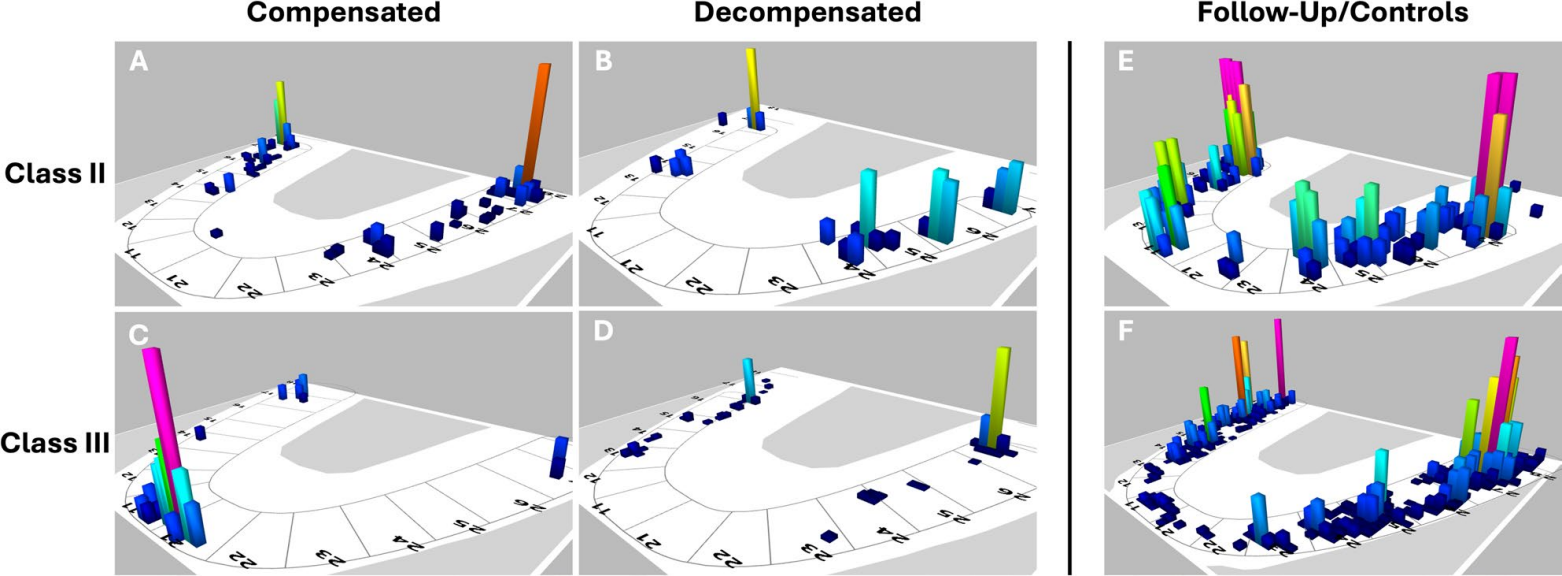
Analysis interface of the T-Scan 9.1 software. Occlusal analysis of the patients. **A** Compensated Class II; **B** Decompensated Class II; **C** Compensated Class III; **D** Decompensated Class III; **E** Follow-up patient; **F** Control

All patients underwent digital occlusal registration using the T-Scan Novus and associated T-Scan 9.1 software (Tekscan Inc., South Boston, Massachusetts, USA; Fig. 2) in an upright sitting position. For registration, patients were asked to bite on the sensor foil in maximal intercuspidal position and to open the mouth again after reaching this position. As occlusal registration was performed in maximal intercuspation, potential functional mandibular shifts were not evaluated separately.

According to Wiechens et al., following occlusal parameters were collected: total tooth contact (TTC), time of occlusion (TOC), occlusal asymmetry (OAS), anterior and posterior tooth contact (ATC and PTC) (Table 3). Occluding teeth were initially recorded in absolute terms and then further categorized into the following groups: posterior teeth (all teeth distal to the lateral incisors, including the second molars) and anterior teeth (mesial to the canines) [21, 22]. Occlusal registration was performed once per participant. In cases where an adequate occlusal record could not be obtained, the procedure was repeated until a stable and reproducible registration was achieved. All occlusal registrations were performed using a standardized protocol by experienced examiners. No formal intra- or inter-examiner reliability testing was conducted.

**Table 3:** Definition and interpretation of the parameters

Occlusal Parameters	Unit	Definition
Total Tooth Contact (TTC)	%	Ratio of all maxillary teeth with antagonistic contact at individual maximum intercuspation, expressed as: (teeth in contact/total teeth) × 100. Interpretation: Higher percentages indicate more complete antagonistic contact.
Time of Occlusion (TOC)	s	Time from initial tooth contact to maximum intercuspation. Interpretation: Shortened time was seen as efficient, lengthened time as inefficient.
Occlusal Asymmetry (OAS)	%	Difference between the more and less force-loaded quadrants. Interpretation: Lower values indicate greater force symmetry across quadrants.
Anterior Tooth Contact (ATC)	%	Percentage of antagonistic tooth contacts within the anterior segment, defined from right to left lateral incisor. Interpretation: Lower percentages indicate reduced functional anterior antagonism.
Posterior Tooth Contact (PTC)	%	Percentage of antagonistic tooth contacts within the posterior segments distal to the lateral incisors. Interpretation: Lower percentages indicate reduced functional posterior antagonism.

### Functional assessment

**Fig. 3:**
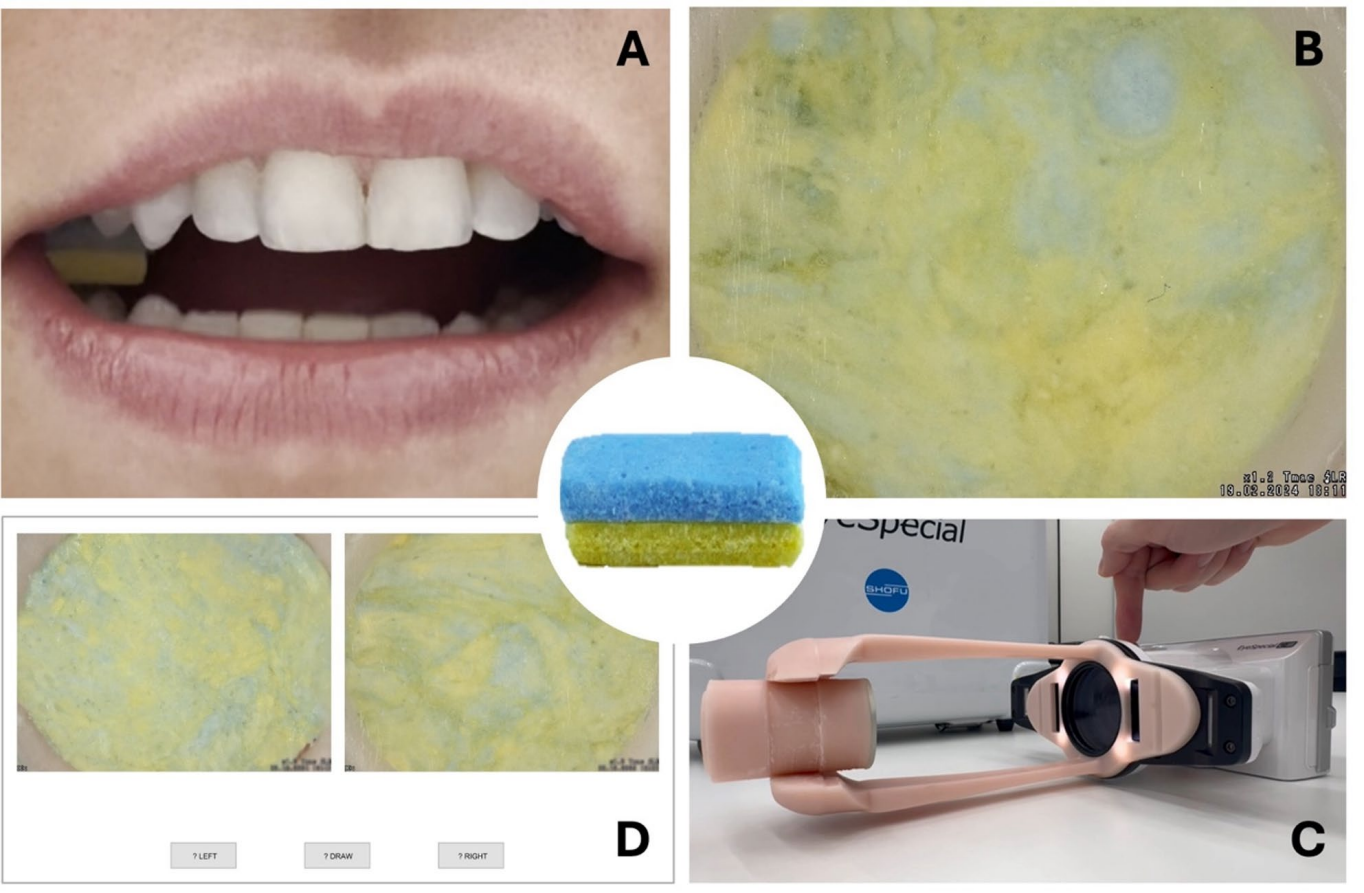
Clinical bolus assessment. Preparation and standardized chewing of a two-coloured gum sample. **A** patient chewing in a circular motion across the dental arch for 20 cycles a strip of yellow and blue chewing gum (middle); **B**, **C** homogenised bolus placed in a 3D-printed photo mount and compressed with a patrix punch prior to overhead photography. **D** Analysis of the chewed bolus for calculating the Bolus BT

Chewing performance was evaluated using a standardized two-color chewing gum test (Fig. 3). For each participant, a bolus was formed from a 21-mm strip of blue and yellow chewing gum (Trident, Mondelez Global LLC, USA). Participants were seated upright and instructed to chew the bolus in a circular motion along the dental arch for 20 cycles. The degree of bolus mixing was quantified using the Bolus BT score [24], an image analysis method described by Mouchoux et al. [24].

This score expresses the probability of the bolus of being rated as better mixed than another one by human experts based on its characteristics (e.g. color range, homogeneity, cluster size), which offers a consistent measure of overall chewing efficiency. The Bolus BT score is a relative measure with a mean of 0. Its range is not fixed and depends on the number of elements included in the calculation and the distribution within the study sample. Higher values indicate a more thoroughly mixed bolus.

### Statistics

A priori sample size calculation was performed in a previous study [21] using G*Power (v. 3.1.9.2, University of Düsseldorf). Assuming a significance level of 0.05, a power of 0.8, and an anticipated drop-out rate of 10%, a required sample size of 14 patients per group was determined.

Statistical analyses were performed using GraphPad Prism 10 (v. 10.2.3, GraphPad Software, USA). All analyses were performed using the Kruskal-Wallis test for independent samples, with the Shapiro-Wilk test showing overall non-normally distributed variables. Additionally, Dunn’s multiple comparisons tests were performed to analyze the differences between the groups. The significance level was set at *p* < 0.05.

Specific measurement parameters were analyzed using descriptive statistics and are reported as medians with interquartile ranges. The tooth contacts were recorded as percentages to account for individual configurations like therapeutic premolar extractions or premolar agenesis, and to allow comparison with fully toothed participants. TTC, OAS, ATC, and PTC are presented as percentages using the associated medians and interquartile ranges with the TOC being rounded to the nearest hundredth of a second.

## Results

Patient-specific data, including key clinical and radiological appearance are summarised in Table 1 and pre-orthognathic parameters of the follow-up cohorts are shown in Table 2.

### Class II malocclusion

In patients with skeletal Class II malocclusion, significant differences in occlusal parameters were observed between non-surgically treated subgroups and controls. TTC was significantly reduced in both dentoalveolarly compensated (64.28%, IQR 39.84) and orthodontically decompensated Class II patients (42.85%, IQR 23.81) compared with Class I controls (100.00%, IQR 0.00) (both *p* < 0.001), with the lowest median TTC observed in the decompensated subgroup. In contrast, no significant difference in TTC was found between the surgically treated follow-up cohort and controls.

TOC was significantly prolonged in both compensated (0.27 s, IQR 0.32) and decompensated Class II patients (0.26 s, IQR 0.14) compared to controls (0.14 s, IQR 0.09) (*p* = 0.016 and *p* = 0.027), whereas TOC values in the follow-up cohort did not differ significantly from those of the control group.

**Fig. 4:**
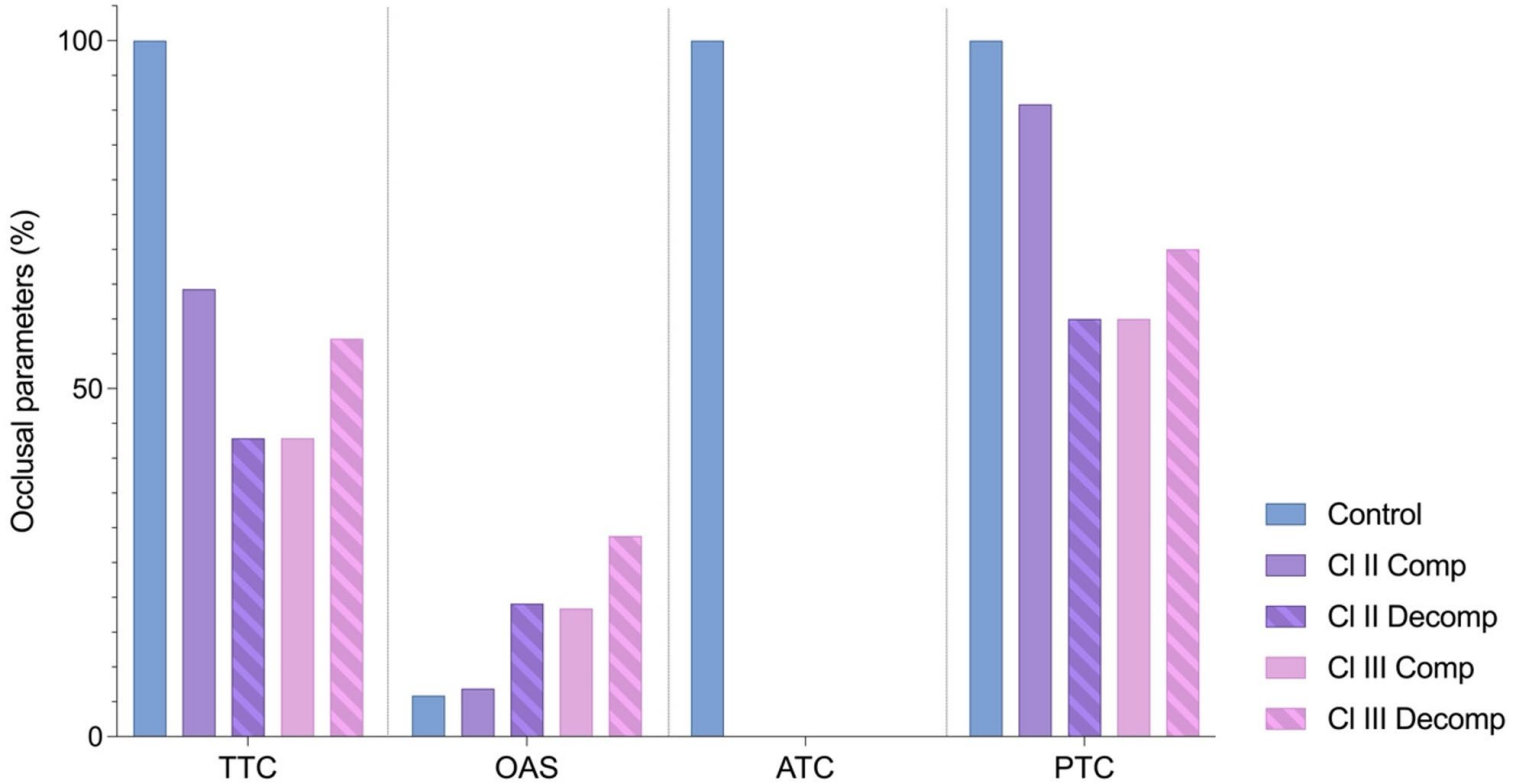
Occlusal parameters (class II and III malocclusion). The abbreviations used are: total tooth contact (TTC), occlusal asymmetry (OAS), anterior tooth contact (ATC) and posterior tooth contact (PTC). The median is reported. Control refers to the control group. Cl II Comp and Cl III Comp refer to Class II and Class III compensated patients, while Cl II Decomp and Cl III Decomp refer to Class II and Class III decompensated patients

**Fig. 5:**
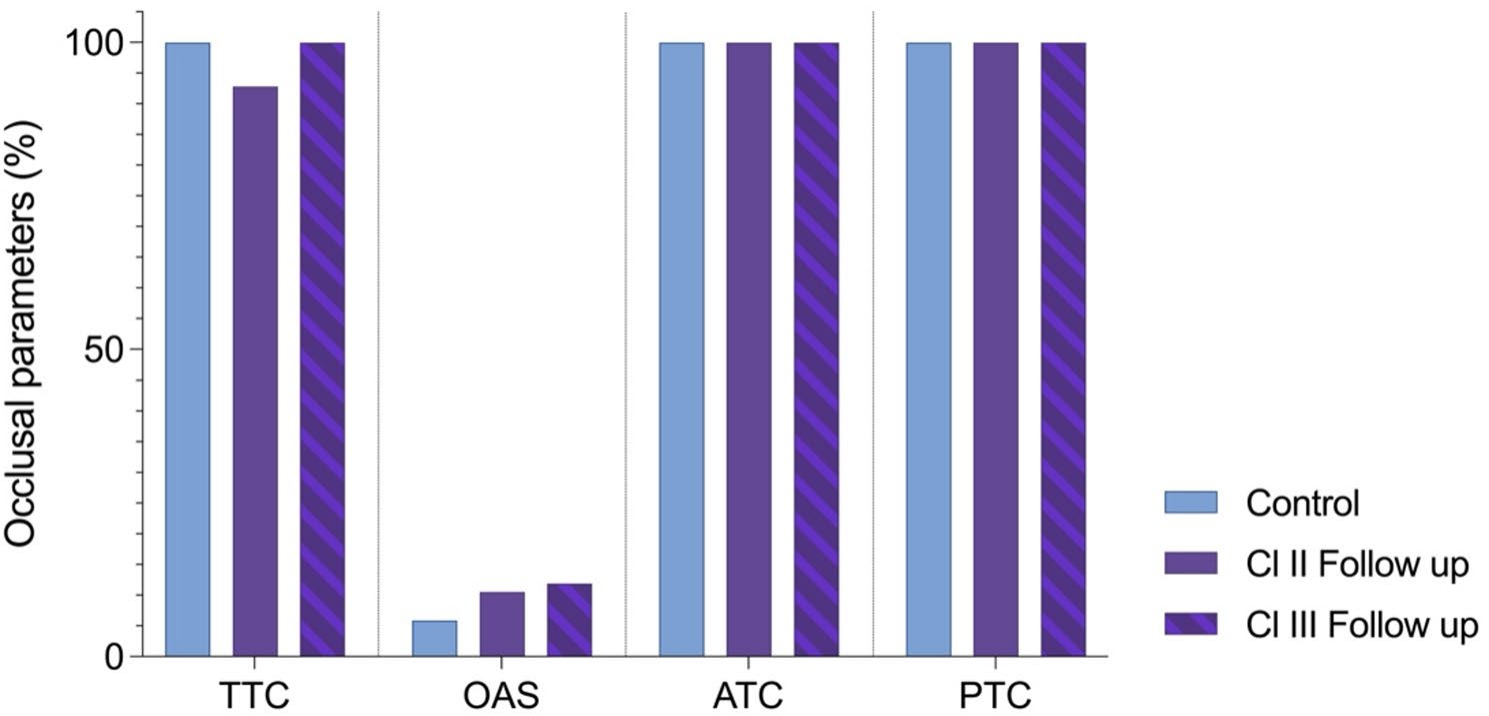
Occlusal parameters (follow-up). The abbreviations used are: total tooth contact (TTC), occlusal asymmetry (OAS), anterior tooth contact (ATC) and posterior tooth contact (PTC). The median is reported. Control refers to the control group, while Cl II Follow up and Cl III Follow up refer to the respective follow-up groups

While OAS showed no significant differences between Class II subgroups and controls, ATC was not evident in both compensated and decompensated Class II patients (both 0.00%, IQR 0.00) and differed significantly from controls (100.00%, IQR 0.00) (both *p* < 0.001). In contrast, ATC in the follow-up cohort was comparable to the controls. PTC was significantly reduced in both non-surgically treated Class II subgroups (compensated: 90,84%, IQR 42.50; decompensated: 60.00%, IQR 32.50) compared with controls (100.00%, IQR 0.00) (*p* = 0.028 for compensated and *p* < 0.001 for decompensated patients), whereas no significant difference was observed in the follow-up cohort (100.00%, IQR 50.00) (Figs. 4 and 5; Tables 4, 5 and 6).

**Table 4:** Descriptive statistics of the occlusal parameters (class II and III malocclusion)

Occlusal Parameters		Controls I (*N* = 20)		Class II (*N* = 40)				Class III (*N* = 40)			
				Compensated (*N* = 20)		Decompensated (*N* = 20)		Compensated (*N* = 20)		Decompensated (*N* = 20)	
	Unit	Median	IQR	Median	IQR	Median	IQR	Median	IQR	Median	IQR
TTC	%	100.00	0.00	64.28	39.84	42.85	23.81	42.85	0.34	57.14	28.57
TOC	s	0.14	0.09	0.27	0.32	0.26	0.14	0.34	0.22	0.32	0.14
OAS	%	5.90	9.60	6.90	23.50	19.10	22.90	18.40	13.95	28.80	23.80
ATC	%	100.00	0.00	0.00	0.00	0.00	0.00	0.00	50.00	0.00	0.25
PTC	%	100.00	0.00	90.84	42.50	60.00	32.50	60.00	38.75	70.00	40.00
Bolus (BT)	-	4.07	12.64	-2.00	4.60	-1.30	3.45	-1.80	12.33	-2.20	5.35

The median and the interquartile range (IQR) are reported

The *abbreviations* used are: *TTC* total tooth contact, *TOC* time of occlusion, *OAS* occlusal asymmetry, *ATC* anterior tooth contact, and *PTC* posterior tooth contact

**Table 5:** Descriptive statistics of the occlusal parameters (follow-up)

Occlusal Parameters		Class II Follow-Up (*N* = 18)		Class III Follow-Up (*N* = 15)	
	Unit	Median	IQR	Median	IQR
TTC	%	92.85	14.29	100.00	7.15
TOC	s	0.14	0.04	0.14	0.10
OAS	%	10.50	8.65	11.90	11.30
ATC	%	100.00	50.00	100.00	0.00
PTC	%	100.00	0.00	100.00	0.00
Bolus (BT)	-	3.25	5.57	1.59	4.03

The median and the interquartile range (IQR) are reported. These post-treatment values of the follow-up cohorts represent measurements obtained 5 years after completion of orthognathic surgery

The *abbreviations* used are: *TTC *total tooth contact, *TOC* time of occlusion, *OAS* occlusal asymmetry, *ATC* anterior tooth contact, and *PTC* posterior tooth contact

**Table 6:** Overall and intergroup comparisons

Occlusal Parameters			Intergroup Comparison					
			Class II			Class III		
	Overall Group Comparison	Compensated vs. Control	Decompensated vs. Control	Follow-Up vs. Control	Compensated vs. Control	Decompensated vs. Control	Follow-Up vs. Control	
U	*p*	corr.*p*	corr.*p*	corr.*p*	corr.*p*	corr.*p*	corr.*p*	
TTC	%	< 0.001***	< 0.001***	< 0.001***	n.s.	< 0.001***	< 0.001***	n.s.
TOC	s	< 0.001***	0.016*	0.027*	n.s.	0.007**	0.009**	n.s.
OAS	%	0.007**	n.s	n.s.	n.s.	n.s	0.006**	n.s.
ATC	%	< 0.001***	< 0.001***	< 0.001***	n.s.	< 0.001***	< 0.001***	n.s.
PTC	%	< 0.001***	0.028*	< 0.001***	n.s.	< 0.001***	< 0.001***	n.s.
BT	-	< 0.001***	0.003**	0.001**	n.s.	< 0.001***	0.003**	n.s.

Corresponding results of the Kruskal-Wallis one-way analysis of variance for the overall comparison between the groups, followed by post-hoc tests for pairwise group comparisons, are presented to analyze differences. The significance level was set at *p* < 0.05, adjusted by Bonferroni correction. Kruskal-Wallis and post-hoc-test

*= *p* < 0.05; **= *p* < 0.01; ***= *p* < 0.001

**Fig. 6:**
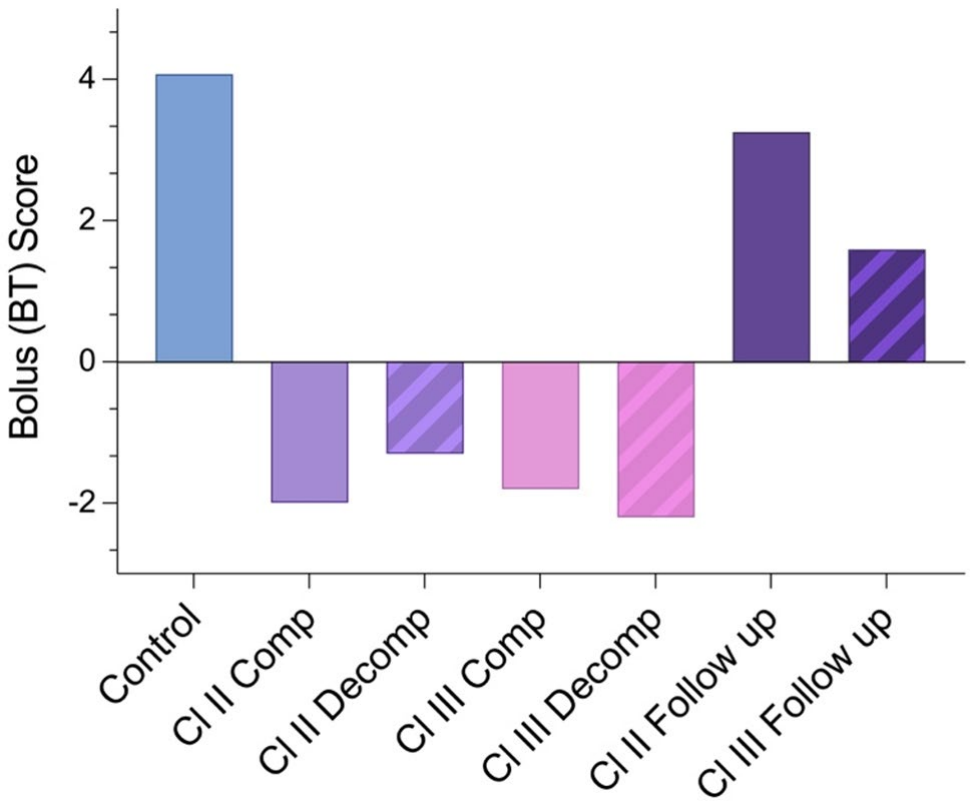
Results of Bolus BT scores across patient groups. Control refers to the control group. Cl II Comp and Cl III Comp refer to Class II and Class III compensated patients, while Cl II Decomp and Cl III Decomp refer to Class II and Class III decompensated patients. Cl II Follow up and Cl III Follow up refer to the respective follow-up groups. The median is reported

Masticatory efficiency (Bolus BT) was significantly lower in both compensated (-2.00, IQR 4.60) and decompensated Class II patients (-1.30, IQR 3.45) compared with controls (4.07, IQR 12.64) (*p* = 0.003 and *p* = 0.001). No significant difference in Bolus BT values was observed between the follow-up cohort (3.25, IQR 5.57) and the controls (Fig. 6; Table 6).

### Class III malocclusion

Similar patterns were observed in patients with skeletal Class III malocclusion. TTC was significantly reduced in both compensated (42.85%, IQR 0.34) and decompensated Class III patients (57.14%, IQR 28.57) compared with controls (100.00%, IQR 0.00) (both *p* < 0.001). In contrast, TTC in the surgically treated follow-up cohort (100.00%, IQR 7.15) did not differ significantly from controls.

TOC was significantly prolonged in both compensated (0.34 s, IQR 0.22) and decompensated Class III patients (0.32 s, IQR 0.14) relative to controls (0.14 s, IQR 0.09) (*p* = 0.007 and *p* = 0.009), while TOC in the follow-up cohort (0.14 s, IQR 0.10) showed no significant difference compared with the controls. OAS was significantly increased in decompensated Class III patients (28.80%, IQR 23.80) compared with controls (*p* = 0.006), whereas no significant differences were detected for compensated patients or the follow-up cohort.

ATC was significantly reduced in both compensated and decompensated Class III patients (both 0.00%; *p* < 0.001). In contrast, ATC values in the follow-up cohort (100.00%, IQR 0.00) were comparable to those observed in Class I controls. PTC was significantly reduced in both non-surgically treated Class III subgroups (compensated: 60.00%, IQR 38.75; decompensated: 70.00%, IQR 40.00) compared with controls (100.00%, IQR 0.00) (both *p* < 0.001), with no significant difference being observed in the follow-up cohort (Figs. 4 and 5; Tables 4, 5 and 6).

Masticatory efficiency (Bolus BT) was significantly lower in both compensated (-1.80, IQR 12.33) and decompensated Class III patients (-2.20, IQR 5.35) relative to controls (4.07, IQR 12.64) (*p* < 0.001 and *p* = 0.003), whereas the follow-up cohort (1.59, IQR 4.03) did not differ significantly from the control group (Fig. 6; Table 6).

## Discussion

The results demonstrate that untreated skeletal Class II and Class III malocclusions are associated with significantly impaired occlusal parameters and reduced masticatory efficiency compared with controls. Both malocclusion groups showed reduced tooth contacts with increased occlusion times. These findings are consistent with previous studies linking reduced antagonistic contacts and increased occlusion time to impaired masticatory performance [2, 14].

The observed longer occlusion times in untreated patients reflect delayed establishment of stable intercuspation, supporting the assumption that occlusal instability contributes to impaired functional efficiency. Digital occlusal analysis using electronic sensors has previously been shown to reliably detect functional disturbances and asymmetries [10, 11]. The findings of the present study further support the diagnostic value of these systems in orthognathic patients, in accordance with recent literature [18].

Although digital occlusal analysis systems such as the T-Scan are widely used to assess occlusal function, the literature shows both strengths and limitations. While several studies have demonstrated acceptable reproducibility and sensitivity for detecting occlusal changes [10, 11], the direct translation of these parameters into functional clinical outcomes is still limited. Sensor-based systems are generally reliable for identifying occlusal contact locations, but their validity for assessing contact intensity as well as exact interocclusal relationships is limited, suggesting they should be used as adjunctive tools rather than replacements for conventional methods [25]. The T-Scan system has been also reported to show higher accuracy and reproducibility for dynamic force and timing measurements, improving diagnostic precision [26]. However, findings remain inconsistent, as some studies report good reproducibility for force and timing, while others question its accuracy in locating occlusal contacts compared with traditional methods [27].

The present study additionally incorporated a standardized bolus-based masticatory performance assessment, allowing functional validation of occlusal findings at the level of actual chewing performance. Bolus-based masticatory assessment confirmed the functional impairments detected by the T-Scan. Standardized two-color chewing gum analysis revealed that both compensated and decompensated Class II and Class III patients had significantly lower Bolus BT scores compared with controls, indicating reduced chewing efficiency and aligning with recent literature [19]. These findings demonstrate that functional deficits extend beyond occlusal contact parameters and are detectable at the level of actual bolus transformation, highlighting the clinical relevance of including bolus analysis in functional assessments.

The SDHue [28] and VOH [29] tests are two established methods for assessing masticatory performance based on hue homogeneity analysis. While their validity has been proven for large differences, their sensitivity have not been established. The variance of the measurements remain quite large at a lower mixing ability [30], a limitation previously anticipated by Lo et al. [31]. Specifically, homogeneity of the Hue can only develop after sufficient mechanical breakdown of bolus clusters, as changes in texture precede the homogenization of the Hue.

To avoid limitations that are unavoidably associated with partial mathematical modelling of the transformation process, the Bolus BT score is based on the Bradley-Terry model derived from pairwise comparisons performed by experts and reliably predicts human ratings. This score is strongly correlated with the SDHue, thereby supporting its construct validity. However, unlike SDHue-based approaches, it does not share the same methodological limitations in its construction and has been shown to demonstrate slightly higher effect sizes for standard measures of masticatory mixing ability.

Importantly, Class II and Class III patients examined at a 5-year follow-up after orthognathic surgery showed occlusal parameters and masticatory efficiency comparable to those of Class I controls. The restoration and maintenance of Class I occlusion in these patients likely contributed to the normalization of masticatory efficiency, indicating a functional correlation between Class I occlusion and enhanced chewing performance. Total tooth contact, occlusion time, contact distribution as well as bolus mixing efficiency did not differ significantly from the controls in both skeletal classes, suggesting that orthognathic therapy can lead to normalization in the long term.

By integrating digital occlusal analysis with standardized bolus-based masticatory assessment, this study provides a more comprehensive evaluation of functional outcomes than approaches focusing only isolated on occlusal parameters. The combined methodology allows functional deficits to be identified not only at the occlusal contact level but also at the level of chewing performance, which is of relevance in the functional assessment of orthognathic patients.

### Limitations

Several limitations should be acknowledged. First, the cross-sectional design does not allow causal inferences regarding the observed differences in occlusal parameters and masticatory performance between the groups. Although long-term follow-up patients were included, functional changes over time cannot be assessed directly.

Second, the monocentric study design may influence occlusal outcomes due to standardized surgical and orthodontic approaches, which limits the generalizability of the findings to other clinical settings and treatment protocols.

Post-treatment cephalometric data were not consistently available for all patients in the follow-up cohort, as the present study primarily focused on long-term functional outcomes rather than radiological reassessment.

Additionally, although digital occlusal analysis and standardized bolus assessment provide objective and reproducible measures, they do not allow differentiation between dentoalveolar adaptations and skeletal influences on occlusal function. Future studies incorporating complementary imaging modalities as well as other longitudinal designs could help to clarifying the relative contribution of dental versus skeletal factors to functional outcomes, provided that such imaging is always clinically justified.

Furthermore, the sample size of the individual subgroups was limited, which may restrict the transferability of subgroup-specific findings.

Finally, while the combined assessment of occlusal characteristics and masticatory performance offers a comprehensive functional evaluation, this study did not assess patient-reported outcomes or direct clinical endpoints such as quality of life or nutritional status, which should be addressed in future research.

## Conclusion

Class II and Class III malocclusions are associated with impaired occlusal parameters and reduced masticatory efficiency, as evidenced by both digital occlusal measurements and bolus analysis. Five years after orthognathic surgery, patients in both skeletal classes showed normalization of occlusal parameters and masticatory performance, with values comparable to untreated controls. Importantly, all follow-up patients exhibited Class I occlusion. With caution, it can be concluded that Class I occlusion is correlated with enhanced masticatory function and associated with significantly higher functional outcomes.

These findings indicate that combined surgical and orthodontic treatment achieves stable, long-term improvements in functional chewing ability, in addition to restoring the skeletal and occlusal dimensions.

## Data Availability

The data underlying this article are available in the article.

## Abbreviations

ATC: Anterior tooth contact
BT: Bolus BT score
OAS: Occlusal asymmetry
PTC: Posterior tooth contact
TOC: Time of occlusion
TTC: Total tooth contact
